# Supplementary material for: Genome-wide identification, characterization and gene expression of BES1 transcription factor family in grapevine (Vitis vinifera L.)
Source: Sci Rep. 2023 Jan 5;13:240. doi: 10.1038/s41598-022-24407-y (PMC9816167; doi:10.1038/s41598-022-24407-y)
Supplement: Supplementary file 3 — Supplementary Information. [file 41598_2022_24407_MOESM3_ESM.zip › Vvi_Atr/Vitis_vinifera.PN40024.v4.dna_sm.toplevel.fa.vs.Amborella_trichopoda.AMTR1.0.dna_sm.toplevel.fa.html/Atr-AmTr_v1.0_scaffold00182.html]

|  |  |  |  |  |  |  |  |  |  |  |  |  |  |
| --- | --- | --- | --- | --- | --- | --- | --- | --- | --- | --- | --- | --- | --- |
| Duplication depth | Reference chromosome | Collinear blocks | | | | | | | | | | | |
| 1 | Atr-ERN16317 |  | Vvi-Vitvi03g00057\_t001 |  |  |  |  |  |  |  |  |  |  |
| 1 | Atr-ERN16318 |  | \| \| |  |  |  |  |  |  |  |  |  |  |
| 1 | Atr-ERN16319 |  | \| \| |  |  |  |  |  |  |  |  |  |  |
| 1 | Atr-ERN16320 |  | Vvi-Vitvi03g00056\_t001 |  |  |  |  |  |  |  |  |  |  |
| 2 | Atr-ERN16321 |  | \| \| |  | Vvi-Vitvi18g01059\_t001 |  |  |  |  |  |  |  |  |
| 2 | Atr-ERN16322 |  | \| \| |  | \| \| |  |  |  |  |  |  |  |  |
| 2 | Atr-ERN16323 |  | Vvi-Vitvi03g04022\_t001 |  | Vvi-Vitvi18g01058\_t001 |  |  |  |  |  |  |  |  |
| 2 | Atr-ERN16324 |  | Vvi-Vitvi03g00054\_t001 |  | \| \| |  |  |  |  |  |  |  |  |
| 2 | Atr-ERN16325 |  | \| \| |  | \| \| |  |  |  |  |  |  |  |  |
| 2 | Atr-ERN16326 |  | \| \| |  | Vvi-Vitvi18g01057\_t001 |  |  |  |  |  |  |  |  |
| 2 | Atr-ERN16327 |  | \| \| |  | Vvi-Vitvi18g01056\_t001 |  |  |  |  |  |  |  |  |
| 2 | Atr-ERN16328 |  | Vvi-Vitvi03g00052\_t001 |  | \| \| |  |  |  |  |  |  |  |  |
| 2 | Atr-ERN16329 |  | Vvi-Vitvi03g01334\_t001 |  | \| \| |  |  |  |  |  |  |  |  |
| 2 | Atr-ERN16330 |  | Vvi-Vitvi03g00051\_t001 |  | \| \| |  |  |  |  |  |  |  |  |
| 2 | Atr-ERN16331 |  | \| \| |  | \| \| |  |  |  |  |  |  |  |  |
| 2 | Atr-ERN16332 |  | \| \| |  | \| \| |  |  |  |  |  |  |  |  |
| 2 | Atr-ERN16333 |  | \| \| |  | \| \| |  |  |  |  |  |  |  |  |
| 2 | Atr-ERN16334 |  | Vvi-Vitvi03g00050\_t001 |  | Vvi-Vitvi18g01055\_t001 |  |  |  |  |  |  |  |  |
| 2 | Atr-ERN16335 |  | \| \| |  | \| \| |  |  |  |  |  |  |  |  |
| 2 | Atr-ERN16336 |  | \| \| |  | \| \| |  |  |  |  |  |  |  |  |
| 2 | Atr-ERN16337 |  | \| \| |  | \| \| |  |  |  |  |  |  |  |  |
| 2 | Atr-ERN16338 |  | \| \| |  | \| \| |  |  |  |  |  |  |  |  |
| 2 | Atr-ERN16339 |  | Vvi-Vitvi03g00049\_t001 |  | Vvi-Vitvi18g01048\_t001 |  |  |  |  |  |  |  |  |
| 0 | Atr-ERN16340 |  |  |  |  |  |  |  |  |  |  |  |  |
| 0 | Atr-ERN16341 |  |  |  |  |  |  |  |  |  |  |  |  |
| 0 | Atr-ERN16342 |  |  |  |  |  |  |  |  |  |  |  |  |
| 0 | Atr-ERN16343 |  |  |  |  |  |  |  |  |  |  |  |  |
| 0 | Atr-ERN16344 |  |  |  |  |  |  |  |  |  |  |  |  |
| 0 | Atr-ERN16345 |  |  |  |  |  |  |  |  |  |  |  |  |
| 0 | Atr-ERN16346 |  |  |  |  |  |  |  |  |  |  |  |  |
| 0 | Atr-ERN16347 |  |  |  |  |  |  |  |  |  |  |  |  |
| 0 | Atr-ERN16348 |  |  |  |  |  |  |  |  |  |  |  |  |
| 0 | Atr-ERN16349 |  |  |  |  |  |  |  |  |  |  |  |  |
